# What do we know about abnormally low prolactin levels in polycystic ovary syndrome? A narrative review

**DOI:** 10.1007/s11154-024-09912-x

**Published:** 2024-10-19

**Authors:** Nicoletta Cera, Joana Pinto, Duarte Pignatelli

**Affiliations:** 1 Faculty of Psychology and Education Sciences, University of Porto, Porto, Portugal; 2 Research Unit in Medical Imaging and Radiotherapy, Cross I&D Lisbon Research Center, Escola Superior de Saúde da Cruz Vermelha Portuguesa, Lisbon, Portugal; 3 Faculty of Medicine, University of Porto, Porto, Portugal; 4 Department of Endocrinology, University Hospital S João and Faculty of Medicine of the University of Porto, Porto, Portugal; 5 I3S Institute for Innovation in Health, Porto, Portugal

**Keywords:** PCOS, Prolactin, PCOS metabolic alterations, Hypoprolactinemia, Hyperprolactinemia

## Abstract

Hyper and hypoprolactinemia seem to be related to the occurrence of metabolic alterations in PCOS patients. In contrast, between significantly elevated and significantly low, prolactin levels seem to be protective against metabolic consequences. In the present review, we found 4 studies investigating hypoprolactinemia in patients with PCOS. We also identified 6 additional studies that reported low levels of PRL in PCOS patients. Although its prevalence is not considered high (13.2–13.9%), its contribution is certainly significant to the metabolic alterations observed in PCOS (insulin resistance, obesity, diabetes mellitus, and fatty liver disease). Dopamine inhibits the secretion of prolactin and GnRH. If dopamine levels are low or the dopamine receptor is less expressed or mutated, the levels of prolactin and GnRH increase, and consequently, LH also increases. On the other hand, hyperprolactinemia, in prolactinomas-typical levels, acting through kisspeptin inhibition causes GnRH suppression and hypogonadotropic hypogonadism. In situations of hypoprolactinemia due to excessive dopamine agonist treatment, dosage reduction is important to minimize the decrease in prolactin levels. Nevertheless, there is a lack of prospective studies confirming these hypotheses, as well as randomized clinical trials with appropriate drugs targeting both hyperprolactin and hypoprolactin in patients with PCOS.

## Introduction

PCOS is considered the most common endocrine disorder in women of reproductive age. PCOS is a disorder associated with neuroendocrine dysfunction and is frequently characterized by increased gonadotropin-releasing hormone (GnRH) pulsatility, luteinizing hormone (LH), and a luteinizing hormone-to-follicle-stimulating hormone (LH/FSH) ratio.

PCOS is characterized by androgen excess, ovulatory dysfunction, and polycystic ovarian morphology (PCOM) [1]. Guidelines require the presence of two or more of the three mentioned criteria for a diagnosis of PCOS [2]. The most common forms of presentation include irregular menstrual cycles and hirsutism [3], but acanthosis nigricans [4] and increased risk of infertility [5] also need to be considered. The worldwide impact of PCOS varies from 5 to 15% (up to 20%) of female patients of reproductive age [1]. Nonetheless, PCOS is not only a multifactor disorder but also a polyhedric syndrome that has several cardiovascular, metabolic, and psychiatric comorbidities that have a negative impact on the quality of life of patients [6]. During the last few years, the role of prolactin (PRL) in several pathological conditions has attracted the attention of the scientific community. The same was true for the effect of PRL on several symptoms and comorbidities of PCOS. Due to its complex nature and the variability in the severity of symptoms among patients with PCOS, there are many areas of uncertainty about a possible link between the levels of serum PRL and this condition. Recently, links between abnormally high serum levels of PRL and PCOS comorbidities have been suggested, mainly regarding the contributions of hyperprolactinemia to infertility and metabolic issues [7].

The prevalence of hyperprolactinemia was estimated to be 4% in a cohort of female blood donors [8], with an estimated incidence rate of 49 per 100,000 persons/year [9].

On the other hand, the impact of abnormally low serum levels of PRL, a condition known as hypoprolactinemia, on PCOS and its related comorbidities and symptoms and the possible role of PRL deficiency in its pathogenesis are even less studied and, in many aspects, are unknown. According to the most recent review [10], hypoprolactinemia is a less known and neglected condition than the widely studied hyperprolactinemia. This is due to its rarity and the absence of patients with significant isolated deficiency. Its deficiency has been observed in puerperal alactogenesis, and in rodent studies, it was found to be related to ovulation impairment and reproductive defects [11].

The present review aimed to characterize the state of the art of studies that have investigated the association between hypoprolactinemia and PCOS. Moreover, the present review takes into account the effect of low levels of PRL and not only true situations of hypoprolactinemia and their effects on both PCOS and related comorbidities.

## Methods

This is not a systematic review, but we performed a systematic search of the principal electronic databases, such as Scopus, Web of Science, PubMed, and Google Scholar. To find the most relevant published studies, we used terms such as “hypoprolactinemia”, “low prolactin”, “low PRL” and “PCOS”, with the Boolean operators AND and OR, with no time limits and written in the English language. Two authors independently assessed the resulting hits for each database search. All the meta-analyses and systematic and narrative reviews were subsequently screened to identify potentially relevant published hits.

Similarly, we also searched previously published studies that investigated low PRL levels or the effect of hypoprolactinemia in other non-PCOS-related pathological conditions to find studies about the association between abnormally low PRL levels and PCOS.

### Previous studies on hypoprolactinemia and PCOS

We found eleven hits published between 1988 and 2024 in English. Among these, we found only four previously published studies that investigated hypoprolactinemia and PCOS [12–15]. The principal characteristics of the abovementioned studies are summarized in Table 1. In a pioneering study, Szilágyi et al. [12], after performing a thyrotropin-releasing hormone (TRH) stimulation test in PCOS patients, reported an exaggerated PRL response in both hypoprolactinemic and normoprolactinemic PCOS patients, with PRL levels > 1100 mU/l and > 2000 mU/l, respectively. In this study, hypothyroidism cannot be assumed to be a possible cause of the low PRL observed in some of the patients with PCOS because both basal and stimulated thyroid-stimulating hormone (TSH) levels were normal [12]. However, according to the authors, PCOS patients react to TRH stimulation independently of the basal PRL level.

**Table 1:** Principal results and characteristics of the studies that investigated hypoprolactinemia in patients with PCOS

Source	Country	Subjects	Age	Design	Assessment	Results
Szilágyi et al., [12]	Hungary	9 hypoprolactinemic PCOS (< 100mU/L) 6 normoprolactinemic PCOS (150–400 mU/L) 6 Controls **(~ 300 μm/L)**	PCOS = 25.2 (18.0–31.0)	Case-control	Basal and stimulated PRL TSH	TSH levels were within normal values. Prolactin responses to TRH were exaggerated in PCOS patients, irrespective of the basal prolactin values.
Kamrul-Hasan et al., [13]	Bangladesh	1039 PCOS: 117 (13.9%) low PRL (< 7 ng/mL) 579 (68.9%) normal PRL (7–25 ng/mL) 144 (17.1%) high PRL (> 25 ng/mL).	Low PRL = 25.0 (20.0–30.0) Normal PRL = 21.0 (18.0–25.0) High PRL = 21.0 (18.0–25.0)	Case‒control	SBP (mmHg) DBP (mmHg) FPG (mmol/L) PG 2 h-OGTT (mmol/L) TG (mg/dL) TC (mg/dL)LDL-C (mg/dL) HDL-C (mg/dL) TT (ng/dL) TSH (µIU/mL)	Prolactin had negative correlations with age, BMI, WC, TG, and the presence of MetS. Prolactin had negative correlations with testosterone and positive correlations with TSH. In multiple regression analysis, prolactin was inversely associated with fasting plasma glucose (FPG) and positively associated with TSH after correcting for age and BM
Aljefri et al., [14]	Saudi Arabia	447 PCOS: Abnormally high PRL, n (%) = 23 (5.1) Normal PRL, n (%) = 323 (72.1) Abnormally low PRL, n (%) = 59 (13.2)	29 (25–34)	Cross-sectional	LH (IU/L) LH/FSH ratio Fasting blood glucose (nmol/L) TSH (mlU/L) Prolactin (ug/L) HbA1c (%) FSH (IU/L) T (nmol/L)	The prevalence of cutaneous manifestations among PCOS patients is relatively high and plays a significant role in making the diagnosis. Therefore, physicians across multiple specialties need to be more aware of the full spectrum of PCOS presentations to alleviate its underdiagnosed status.
Ben Salem et al., [15]	Tunisia	PCOS: 73.1 ± 11.7 (mU/L) Controls: 148.8 ± 9.4 (mU/L)	PCOS: 29.8 ± 0.4 Controls: 33.5 ± 0.5	Cross-sectional		Women with PCOS have higher BMI than controls (*P* < 0.0001; 28.4 ± 0.7 vs. 23.1 ± 0.2 kg/m2), higher random glycemia (*P* < 0.0001, 7.9 ± 0.2 vs. 4.5 ± 0.1 mmol/L), fasting insulin (*P* = 0.01, 15.7 ± 1.2vs 7.7 ± 0.4 mU/L), levels of total testosterone (*P* < 0.0001, 2.9 ± 0.2 vs. 1.0 ± 0.1 noml/L) and of plasma triglycerides (TG) (*P* = 0.0001, 1.7 ± 0.1 vs. 1.0 ± 0.1). Prolactin levels in plasma were lower in women with PCOS compared with controls (*P* = 0.0001, 73.1 ± 11.7 vs. 148.8 ± 9.4)

In a retrospective study, Kamrul-Hasan and Aalpona [13] reported 117 patients with hypoprolactinemia, with a sample composed of 840 PCOS patients. In the hypoprolactinemic PCOS group, significant negative correlations were found between PRL levels (< 7 ng/mL) and metabolic blood indices, such as abnormal glucose tolerance (AGT), fasting plasma glucose (FPG), plasma glucose 2 h after the oral glucose tolerance test (PG 2 h-OGTT), and dyslipidemia, and a weak positive correlation with total testosterone (TT) was detected. This study highlighted the role of low PRL levels in metabolic disorders associated with PCOS. In contrast, PRL > 25 ng/mL and up to 100 ng/mL did not negatively correlate with metabolic indices in PCOS patients.

Similarly, Ben-Salem et al. [15] reported that PCOS patients with hypoprolactinemia had lower levels of PRL and a higher BMI, and similar results were observed, with higher random glycemia, fasting insulin, total testosterone, and plasma triglyceride levels in PCOS patients with hypoprolactinemia than in controls (Table 1). Curiously, this study revealed a correlation between PRL and VEGF, confirming previous reports of a possible proangiogenic effect of PRL. Angiogenesis has also been implicated in disorders of anovulation, subfertility, and other pathogenic conditions, such as endometriosis. These findings may suggest the possible regulation of the prolactin secretion pathway through VEGF or a stimulatory effect of PRL on VEGF production. Low prolactin levels are associated with low angiogenesis and thereby poor follicle development and anovulation.

Aljefri et al. [14] studied a sample of 447 PCOS patients and reported that 59 women (13.2%) had abnormally low PRL. However, they reported that PRL was not associated with the cutaneous manifestations of PCOS (such as acne, hirsutism, and androgenic alopecia). Notably, among the studies mentioned above, only the first [12] investigated hypoprolactinemia in women with PCOS for this purpose, whereas the other studies only indirectly reported PRL levels in women with PCOS.

Since the interest of this review is in the deficiency of PRL, we found six published studies that reported low levels of PRL in PCOS patient subgroups (Table 2) [16–21]. According to these studies, low levels of PRL in patients with PCOS were predominantly related to an unfavorable metabolic profile. Albu et al. [16] reported that PCOS patients with an unfavorable metabolic profile had low PRL. Nonetheless, according to the authors [16], the metabolic effects of low PRL were probably not direct but rather the result of an interplay among prolactin, adiposity, and insulin resistance. In PCOS patients, PRL levels are negatively associated with the quantity and function of adipose tissue and are positively correlated with the serum adiponectin level [16].

**Table 2:** Principal characteristics of the studies that reported low levels of PRL in PCOS patients

Source	Country	Subjects	Age	Design	Assessment	Results
Albu et al., [16]	Romania	322 PCOS	M (SD) = 24 (7)	Case-control	Fasting glycemia (mg/dL) 2 h-glycemia (mg/dL) Fasting insulin (µUI/mL) 2 h-insulin (µUI/mL) HOMA-IR TT (ng/mL) SHBG (nmol/L) Prolactin (ng/mL) Adiponectin (mg/L) Leptin (ng/ml) LH (mUI/L) FSH (mUI/L) E2 (pg/mL) TSH (µUI/mL) FAI	In PCOS patients, serum prolactin level was related to adipose tissue quantity and function, and adiponectin was a possible mediator of this relationship. Low serum prolactin levels were associated with an unfavorable metabolic profile, but this association seemed to be due to the complex interplay among prolactin, adiposity, and insulin resistance rather than to a direct metabolic effect of prolactin.
Yang et al., [17]	China	2,052 PCOS 9,696 patients with tubal infertility (non-PCOS)	20–40 years	Retrospective study	Serum PRL, FSH, LH, T, E_2_ TSH, FT3, FT4, fasting plasma lipid, fasting plasma glucose (FPG), liver function, thyroid hormone	PRL levels were significantly lower in PCOS patients than controls over all age groups (*p* < 0.05). In the PCOS patients, serum PRL was significantly and positively correlated with FPG, serum TSH, and serum FT4, and significantly and negatively correlated with LH, LH/FSH, TC, TG, LDL-C, AST, ALT, γ-GGT, FT3, and FT3/FT4 (*p* < 0.05 or 0.01). After adjusting for age and body mass index (BMI), serum PRL was positively correlated with FPG, TSH, and FT4, and negatively correlated with LH and LH/FSH.
Yang et al., [18]	China	792 PCOS 700 non-PCOS infertile women		Retrospective Cross-sectional study		PCOS, prolactin levels were lower compared to those without PCOS, even after considering age and weight. Prolactin was higher when HDL-C was high but lower with increasing age, weight, and markers of insulin resistance and liver health. After adjusting for age and weight, prolactin decreased with higher levels of certain hormones and insulin resistance markers.
Saei et al., [19]	Iran	8551 PCOS 13,737 Controls		Meta-Analysis/Systematic Review	-	Subgroup analysis of PRL levels according to the continent of origin showed significantly higher PRL levels among Eurasian PCOS patients compared to the control; this difference was not statistically significant in the subgroups of women from Asia, Europe, and South America. Moreover, PCOS patients in the African population have significantly lower PRL levels.
Ponce et al., [20]	Mexico	Normal BMI < 25, *n* = 9 Overweight BMI 25–29.9, *n* = 16 Obese BMI ≥ 30, *n* = 15		Observational study		Lower PRL serum levels are associated with adipocyte hypertrophy, in visceral but not in subcutaneous fat, and with a higher HOMA-IR. Furthermore, low systemic PRL levels together with high waist circumference predict an elevated HOMA-IR whereas low serum PRL values in combination with high blood glucose predict visceral adipocyte hypertrophy. In agreement, visceral fat from insulin-resistant subjects shows reduced expression of prolactin receptors. However, there is no association between PRL levels and obesity or signs of metabolic syndrome.
Glintborg et al., [21]	Denmark	1007 PCOS 116 Controls	PCOS: 30 (23–36) Controls: 28 (24–37)	Retrospective cross-sectional study	Serum PRL Ferriman-Gallwey score BMI waist circumference blood pressure sex hormones fasting lipids insulin glucose transvaginal ultrasound oral glucose tolerance adrenocorticotrophic hormone	Prolactin levels were significantly lower in patients versus controls; median (quartiles) prolactin levels were 7 (5–10) versus 9 (7–13) µg/l (*P* < 0.001). In the patient population prolactin levels were inversely associated with age, smoking status, waist circumference, total cholesterol, triglyceride, and low-density lipoprotein (LDL) and positively associated with high-density lipoprotein, estradiol, total testosterone, dehydroepiandrosterone sulfate, 17-hydroxyprogesterone and cortisol levels. In multiple regression analyses, prolactin was inversely associated with LDL and positively associated with estradiol, 17-hydroxyprogesterone, and cortisol after correcting for age, BMI, and smoking status in patients with PCOS.

On the other hand, other studies associated dyslipidemia and metabolic deficits with low levels of PRL in individuals with PCOS [17–18, 20–21]. Interestingly, in a meta-analysis [19], Saei reported that PRL levels were lower in women with PCOS from Africa than in women from Eurasia.

### Prolactin: secretion, function, and dysfunction

Prolactin is a polypeptide hormone that is mainly secreted in the anterior pituitary gland but is also produced in several other body tissues, including the nervous system, uterus, and epithelial mammary cells [22]. Dopamine secreted by hypothalamic neurons is the principal regulatory signal of PRL secretion. The three hypothalamic dopaminergic neuronal populations, tuberoinfundibular, tuberohypophyseal, and periventricular hypophyseal dopaminergic neurons, regulate PRL secretion [23]. The dopamine receptor DRD2 was also found to regulate and suppress PRL secretion. In particular, the lactophoric secretion of prolactin is inhibited by dopamine secreted in the tuberoinfundibular neurons of the hypothalamus through the dopamine 2 receptor, which represents an important tone regulator of lactophoric action [24]. Conversely, the stimulation of PRL secretion is mediated by thyrotropin-releasing hormone (TRH) and 17β-estradiol through actions on the pituitary gland and hypothalamus.

Over the last few years and after extensive research, prolactin has emerged as a modulator of many functions of several body tissues. PRL influences several physiological and pathophysiological functions, such as metabolism [25, 26], behavior and emotion [27, 28], and reproduction [29, 30].

Importantly, PRL was found to be synthesized at extrapituitary sites in which the hypothalamus does not exert its control [31]. Among these sites, human ovarian tissue represents both a target of the pituitary-secreted PRL and a source of in situ PRL [32, 33, 34]. Due to its mode of action, PRL can be classified as a circulating hormone or an autocrine or paracrine factor [35].

Prolactin stimulates proliferation and survival and improves the quality of pancreatic beta cells, increasing insulin secretion and sensitizing the liver to insulin [18]. Prolactin can also affect metabolic homeostasis and glucose and lipid storage by regulating key enzymes and transporters related to glucose and lipid metabolism in target organs.

The biological effect of PRL is mediated by the PRL receptor (PRLR), a member of the cytokine receptor superfamily, which is present in almost all tissues [36]. Several isoforms have been identified that differ in length and the sequences of their intracellular domains, such as different amino acid sequences [37, 38]. In particular, three principal isoforms of PRLR have been characterized in both humans and animals: long, medium, and short. The long isoform is expressed in several tissues, including the ovaries, and plays a relevant role in growing follicles [39].

Insights into the role of PRL were obtained from studies using PRL-/- and PRLR-/- mouse models. Compared with PRLR+/+ female mice, PRLR-/- female mice are described as completely infertile; however, they have normal ovaries and no differences in follicular development, ovulation, or the fertilization rate. The ovulation rate is not different between PRLR+/+ and PRLR−/− mice, and the corpus luteum even begins to form, but an elevated level of apoptosis and extensive inhibition of angiogenesis occur during the luteal transition in the absence of PRL signaling [32]. Therefore, PRL seems to be fundamental for corpus luteum development through its receptor and the luteinization mechanism mediated by the luteinizing hormone [32].

Hyperprolactinemia affects 90 per 100,000 women and is the most common endocrine disorder of the hypothalamic‒pituitary axis [40]. Chronic excess PRL is a widely studied condition, and in addition to its classic symptoms of galactorrhea and interference with the normal menstrual cycle, it is associated with an increased risk of insulin resistance, prediabetes, dyslipidemia, obesity, and endothelial dysfunction [41, 42].

DRD2 receptor agonists, such as cabergoline, which are normally used to inhibit the pituitary production of PRL and lactophoric action [43–45], can counteract the cardiometabolic effects of hyperprolactinemia. However, high levels of PRL can be relevant to some human functions. During the early stages of pregnancy, elevated prolactin levels, with a decrease in dopamine release, are required to stimulate the hormonal ovary pathways to prepare the endometrium for the implantation of a fertilized oocyte [46].

Dopamine neurons that inhibit the production and release of PRL show highly stereotyped oscillatory activity. This oscillation, which warrants PRL release, appears to be regulated by the transient receptor potential (TRP) channel (TRPC5), which is a member of the TRPC subfamily of Ca2 + channels [47, 48]. The pituitary mechanisms underlying PRL release can directly modulate the cycle phase duration and affect the pregnancy rate. In a recent study, Blum et al. [49] reported that mutated TRPC5-deficient female mice had all phases of the reproductive cycle, with irregularities and signs of oligo-ovulation, less frequent oestrus, and longer phases of diestrus, together with hypoprolactinemia. Nevertheless, further studies on TRPC5 gene mutations in humans and their relationship with prolactin deficiency are needed [49].

In contrast to hyperprolactinemia, hypoprolactinemia, which is characterized by abnormally low serum PRL levels, is rare. Due to its rarity, this pathological condition has not been widely studied, and the underlying mechanisms and possible negative effects on human functions are unclear.

In a pioneering study by Garcea in 1983 [50], both iatrogenic hyper and hypoprolactinemia induced by metoclopramide and bromocriptine, respectively, caused luteal insufficiency with decreased progesterone levels compared with those in normoprolactinemic controls. However, these two abnormal PRL conditions did not affect 17β-estradiol or luteinizing hormone levels or follicular maturation. Indeed, the secretion of progesterone in granulosa cells may be affected by both abnormally low and high PRL serum levels [51].

Women with hyperprolactinemia have low sexual desire and arousal, impaired vaginal lubrification, and difficulty reaching orgasm, as measured by the female sexual functioning index (FSFI) [52]. High levels of PRL result in impaired sexual drive [53], whereas low PRL levels, as studied in some PCOS series, are associated with orgasm difficulties [54]. In a group of normal cycling premenopausal women affected by iatrogenic cabergoline-induced hypoprolactinemia, low PRL disturbed libido, sexual arousal, and mood, resulting in mild depressive symptoms. Like abnormally high PRL, low PRL also affects sexual dimensions in women. Nonetheless, hyperprolactinemia affects more sexual domains, from the desire to the orgasm, whereas in hypoprolactinemia, the negative effects involve only a few sexual dimensions [55].

In a recent study [44] comparing two groups of women, one with abnormally high and one with abnormally low PRL levels, together with a third normal-PRL group, women affected by iatrogenic hypoprolactinemia showed a change in carotid intima-media thickness (CIMT), a marker of early atherosclerosis related to cardiovascular morbidity and mortality, independent of other conventional risk factors [56]. These results are similar to those obtained in previous studies [43, 57, 58]. According to Krysiak [44], young premenopausal women with abnormally low or abnormally high PRL levels may be predisposed to cardiovascular and metabolic disorders during aging. In this way, there seems to be an inverse U-shaped relationship between PRL concentration and cardiovascular and metabolic risk.

## Discussion

Prolactin has many functions in humans, ranging from lactation to reproductive processes, neuroprotection, homeostasis, immunoregulation, and orgasm. Prolactin was also shown to stimulate β-cell proliferation, insulin production, and glucose-dependent insulin secretion [59]. By acting through hepatic PRL receptors, prolactin was demonstrated to reduce liver steatosis [60].

Prolactin is produced in pituitary lactotrophic cells and many other tissues, such as the brain, mammary glands, uterus, and lymphoid cells. The hypothalamus tonically inhibits PRL secretion through the secretion of dopamine.

Hyperprolactinemia is one of the most prevalent pituitary disorders. Despite occurring in both sexes, it is much more common in females. Its main symptoms include galactorrhea, menstrual irregularities, infertility, and sexual dysfunction. Importantly, it should be carefully studied to exclude iatrogenic causes as well as tumors that are the most frequent type of tumors of the pituitary gland.

Hypoprolactinemia is associated with agalactia, low levels of IGF-1, elevated levels of triglycerides and low HDL cholesterol levels, obesity, and insulin resistance, all of which are associated with metabolic syndrome, type 2 diabetes mellitus, NAFLD, and sexual dysfunction [10]. The effect of prolactin on sexual dysfunction may be influenced by increased dopaminergic tone [61]. The recognition of the role played by low prolactin levels in the context of sexual dysfunction in both men and women is highly important [62, 63].

Among the causes of hypoprolactinemia are the use of dopamine agonists such as bromocriptine or cabergoline, surgical intervention in the pituitary gland, Sheehan syndrome, traumatic injury, genetic deficiency of lactotroph cell development, and inflammatory or autoimmune hypophysitis.

In the context of PCOS, the diagnosis of the most important prolactin pathologies also has to be excluded. Nonetheless, otherwise, there may be situations in which prolactin dynamics are altered and may influence the symptoms as well as the outcomes of this disease. HPRL and hypoprolactinemia are associated with PCOS, and most frequently, studies have reported high PRL levels.

The possibility of a causal link between hyperprolactinemia and PCOS is still not understood. Since these two diseases are very prevalent, their cause-effect relationships have never been confirmed [7]. One possible explanation for the presence of hyperprolactinemia (in any situation) may simply be the result of an inaccurate method of dosage that does not exclude the presence of macroprolactinemia. This was certainly the case in many older studies that did not consider this limitation in the methodology. Another limitation of older studies is that they did not use the modern standardized criteria for diagnosing PCOS. The prevalence of hyperprolactinemia in patients with PCOS reported in studies after the utilization of the Rotterdam criteria was 11.9% [7].

One possible explanation for the connection between HPRL and PCOS could be a decrease in dopaminergic tone, which is responsible for high levels of both LH and PRL [64–66]. Neuroendocrine changes in dopaminergic and serotoninergic systems related to stressful life events can result in functional HPRL [67, 68]. Compared with non-PCOS women, PCOS women have an increased clinical prevalence of depression, anxiety, and other forms of perceived stress [69–71]. However, some patients tend to perceive less stress, resulting in a different or no neuroendocrine dysregulation of the PRL.

Functional hypothalamic amenorrhea (FHA) is defined as the absence of menstrual cycles caused by suppression of the hypothalamic‒pituitary‒ovarian (HPO) axis without organic causes and is characterized by the presence of polycystic ovary morphology (PCOM). It is potentially reversible and is considered a consequence of stress, weight loss, and physical exercise [72, 73]. However, FHA patients have lower levels of PRL than PCOS patients do [74, 75]. For this reason, it could be plausible to hypothesize that stress plays a role in the neuroendocrine regulation of PRL. Therefore, further studies are needed to disentangle the role of stress in the neuroendocrine regulation of PRL in PCOS.

Notably, dopamine, which inhibits PRL secretion, is also antagonistic to GnRH release and pulsatility [65, 76, 77]. Dopamine infusions have been shown to induce a significant reduction in LH levels in humans, and bromocriptine, a dopamine agonist, has demonstrated efficacy in reducing LH/FSH ratios and restoring ovarian function in women with PCOS [64, 78].

Dopamine exerts its effects through dopamine receptors. Dopamine receptor 2 (DRD2) has been shown to mediate dopamine-mediated inhibition of both GnRH and prolactin secretion. Recently, DRD2 was identified as a gene contributing to PCOS development (polymorphisms in DRD2 may predispose individuals to the development of PCOS), and concomitantly, DRD2 was reported to increase the risk for type 2 diabetes and depression, both of which can coexist with PCOS [79, 80]. In 212 Italian families, five novel variants were found to be significantly linked to the risk of PCOS [69]. Therefore, DRD2 may be a risk gene in PCOS [81].

Relatedly, in PCOS rat models, a reduction in dopamine levels or DRD2 expression was observed [82]. DRD2 is thought to play an important role in the metabolic phenotypes associated with PCOS. In previous animal studies, obese mice with reduced DRD2 activation developed insulin resistance, which was attenuated by DRD2 agonist treatment [81, 83, 84].

Several authors have called attention to alterations in the levels of prolactin, including both low and elevated prolactin, in patients with PCOS. It has been postulated that prolactin may contribute to metabolic protection (PRL levels that are normal or above normal) or to the metabolic alterations that usually accompany PCOS (very low or very high PRL levels). Prolactin concentrations between 25 and 80 ng/ml seem to have positive effects on human metabolism [85].

On the basis of these conclusions, the present review aimed to analyze what has been learned about the presence of hypoprolactinemia in patients with PCOS, a situation that is less often reported or recognized but is not as rare as our review of the literature seems to confirm. Indeed, abnormally low PRL circulating levels in PCOS patients were found to be associated with cardiovascular and metabolic complications, rather than being related to other symptoms or comorbidities. Among the four studies that reported abnormally low PRL values in PCOS patients, two reported significantly increased values in metabolic indexes in PCOS patients with hypoprolactinemia compared with normoprolactinemic PCOS patients and healthy controls [13, 15]. This finding is in line with previous studies that reported the detrimental effects of abnormally low PRL on cardiometabolic health in women without PCOS [20, 86, 87]. It is plausible that altered levels of PRL can increase the incidence of metabolic syndrome, regardless of the presence of PCOS.

No studies reported a specific association with mood disorders, alterations in sleep, or hyperandrogenism.

Several cutoff values to define hypoprolactinemia have been used in different studies (PRL < 1,8 ng/ml [88], PRL < 5 ng/ml [89], or PRL < 4 ng/ml) [90]. Toledano et al. [91] defined hypoprolactinemia as mild if prolactin levels were between 3 and 5 ng/ml and severe if prolactin levels were < 3 ng/ml. More recently, Macotela et al. [85] defined hypoprolactinemia less than 7 ng/ml as the level associated with metabolic abnormalities. Only recently have new cutoff values been suggested for men and women via the TRH stimulation test [92], which assesses PRL responses at 20 and 60 min after IV administration of 200 µg TRH in 48 patients with panhypopituitarism and 20 healthy controls. In the healthy control group, the cutoff was considered the fifth percentile peak after TRH testing for a sufficient PRL response, with a value of 41.6 ng/mL in women. In normoprolactinemic and hypoprolactinemic PCOS women, Szilágyi et al. [12] reported an increase of 8–12-fold at 30 min after TRH stimulation testing. The increase in PRL in women with PCOS might be caused by the stimulatory effect of estrogen on lactotrophs [12]. However, only this study [12] used TRH testing to study the PRL response in a limited sample of PCOS patients affected by hypoprolactinemia, without assessing the response at different time points [93].

A search of the literature revealed that the negative metabolic effects of prolactin follow a U-shaped pattern of risk, with the highest rates being associated with higher and lower levels of this hormone [16–18, 21, 25, 26, 35, 85, 94]. In contrast, in the interval between these two extremes, prolactin seems to have a protective effect against the development of insulin resistance and consequently diabetes mellitus [16, 95, 96]. Normal levels of prolactin have been shown to stimulate the proliferation and survival of beta cells [97, 98]. A moderate increase in prolactin is associated with a decreased incidence of diabetes mellitus [99, 100]. In addition, it has been reported to prevent fatty liver disease (NAFLD) [100].

**In conclusion**, in women with PCOS, hypoprolactinemia is associated with metabolic dysfunction and complications, such as an association with the risk of insulin resistance and consequently with the development of diabetes mellitus and cardiovascular diseases [25, 95, 96].

**Take-home messages**: We should pay more attention to prolactin and the presence of alterations in prolactin levels in patients with PCOS.

- If these are elevated, it is important to exclude and treat the specific causes of the augmentation.
- If these are very elevated or abnormally low, one can expect important metabolic consequences.
- In the interval between significantly elevated and significantly low levels, prolactin seems to protect against the metabolic consequences that affect PCOS.
- In the case of hypoprolactinemia, treatment with dopamine agonists aiming to reduce prolactin levels regardless of the cause should be reconsidered, and the dosage that is being employed should be reduced so that the expected result is obtained without excessively reducing the levels of prolactin [26, 44].
- Dopamine inhibits the secretion of prolactin and GnRH [82]. Up to a certain amount it can prevent the elevation of LH, thus resulting in a lower LH/FSH ratio.
- In contrast, if dopamine levels are low or the dopamine receptor is less expressed or mutated, the levels of prolactin and GnRH increase, and consequently, LH also increases [76, 101, 102].
- However, the existing literature makes it difficult to draw back recommendations about the detrimental effects of hypoprolactinemia in PCOS.

Although hypoprolactinemia is a rare condition, only very few studies have reported low levels of PRL in PCOS. Most of the studies reported the presence of cases of PCOS in which low or abnormally low PRL levels were observed. However, none of the studies discussed here reported a systematic assessment of this condition in a reliable sample of women affected by PCOS, and none of the studies were designed to address this specific deficiency.

For future consideration, it is imperative to perform long-term prospective studies on the effects of prolactin levels on the metabolic consequences of PCOS. Additionally, randomized clinical trials lowering dopamine tonus in PCOS patients may be needed to treat hypoprolactinemia.

## Data Availability

No datasets were generated or analysed during the current study.

## Abbreviations

PRL: Prolactin
PCOS: Polycystic ovary syndrome
PCOM: Polycystic ovarian morphology
LH: Luteinizing hormone
FSH: Follicle stimulating hormone
GnRH: Gonadotropin-releasing hormone
TRH: Thyrotropin-releasing hormone
TSH: Thyroid-stimulating hormone
AGT: Abnormal glucose tolerance
FPG: Fasting plasma glucose
PG 2 h-OGTT: Plasma glucose 2 h after oral glucose tolerance test
TT: Total testosterone
DRD2: Dopamine receptor 2
NAFLD: Nonalcoholic fat liver disease
